# Mesenchymal stem cell spheroids alleviate neuropathic pain by modulating chronic inflammatory response genes

**DOI:** 10.3389/fimmu.2022.940258

**Published:** 2022-08-08

**Authors:** Nayeon Lee, Gyu Tae Park, Jae Kyung Lim, Eun Bae Choi, Hye Ji Moon, Dae Kyoung Kim, Seong Min Choi, Young Cheol Song, Tae Kyun Kim, Jae Ho Kim

**Affiliations:** ^1^ Convergence Stem Cell Research Center, Medical Research Institute, Pusan National University, Yangsan, South Korea; ^2^ Department of Physiology, School of Medicine, Pusan National University, Yangsan, South Korea; ^3^ Department of Anesthesia and Pain Medicine, Pusan National University Yangsan Hospital, Yangsan, South Korea

**Keywords:** mesenchymal stem cells, 3D spheroids, chronic inflammatory response genes, neuropathic pain, chronic constriction injury

## Abstract

Chronic neuropathic pain is caused by dysfunction of the peripheral nerves associated with the somatosensory system. Mesenchymal stem cells (MSCs) have attracted attention as promising cell therapeutics for chronic pain; however, their clinical application has been hampered by the poor *in vivo* survival and low therapeutic efficacy of transplanted cells. Increasing evidence suggests enhanced therapeutic efficacy of spheroids formed by three-dimensional culture of MSCs. In the present study, we established a neuropathic pain murine model by inducing a chronic constriction injury through ligation of the right sciatic nerve and measured the therapeutic effects and survival efficacy of spheroids. Monolayer-cultured and spheroids were transplanted into the gastrocnemius muscle close to the damaged sciatic nerve. Transplantation of spheroids alleviated chronic pain more potently and exhibited prolonged *in vivo* survival compared to monolayer-cultured cells. Moreover, spheroids significantly reduced macrophage infiltration into the injured tissues. Interestingly, the expression of mouse-origin genes associated with inflammatory responses, Ccl11/Eotaxin, interleukin 1A, tumor necrosis factor B, and tumor necrosis factor, was significantly attenuated by the administration of spheroids compared to that of monolayer. These results suggest that MSC spheroids exhibit enhanced *in vivo* survival after cell transplantation and reduced the host inflammatory response through the regulation of main chronic inflammatory response-related genes.

## Introduction

Peripheral neuropathic pain is a complex, chronic, and debilitating condition that severely worsens a patient’s quality of life (1). Chronic peripheral neuropathic pain is caused by pathological changes or diseases of the peripheral somatosensory nervous system (2), and is characterized by neuropathic pain such as hyperalgesia (painful stimuli) or allodynia (painless stimuli) (3, 4). Although neuronal processes most likely trigger the onset of neuropathic pain, accumulating evidence suggests the involvement of immune cells and chronic inflammation in the induction and maintenance of neuropathic pain (5). Several pharmacological treatment approaches have been introduced; however, despite these effects of pain relief, chronic peripheral neuropathic pain does not respond to conventional drug treatments (6).

Stem cells have drawn considerable attention in the therapy of chronic peripheral neuropathic pain (7). Mesenchymal stem cells (MSCs) represent a good source for therapy of various diseases and regeneration of injured tissues through direct differentiation into tissue-type cells or paracrine functions, including pro-angiogenesis, anti-apoptosis, and immunomodulatory effects (8–10). However, the clinical application of MSCs has been hampered by the poor survival and therapeutic efficacy of transplanted cells. MSC spheroids or aggregates, which are formed by three-dimensional (3D) suspension culture, not only enhance the survival of administered MSCs but also better mimic the physiological microenvironment compared to two-dimensional (2D) monolayer-cultured MSCs (11–15). Accordingly, conditions with cell-to-cell interaction and cell-extracellular matrix can be used to preserve viable cells throughout cell transplantation and repair the pain response. However, it has not been explored whether MSC spheroids can affect the survival and therapeutic efficacy of MSCs in chronic peripheral neuropathic pain.

The chronic constriction injury (CCI) model is one of the most frequently used models for studying neuropathic pain. The CCI model is based on the unilateral loose ligation of the sciatic nerve, which corresponds to the pathophysiological properties of chronic neuropathic pain in humans (16). Ligation knots are placed on the sciatic nerve to induce chronic nerve injury (17–19). However, some studies reported the alleviation of symptoms after 2-4 weeks (20), and Urban et al. reported that they rarely observed signs of pain (21). Therefore, it is necessary to perform compression tests on the ligations before applying the CCI model to avoid inconsistencies in the results.

In this study, we aim to demonstrate the MSC implantation in 3D spheroid form can effectively relieve injured pain in the CCI model, and to explore the main mechanisms that effectively respond to inflammatory signaling by improving the survival of the engrafted MSCs. We believe that this strategy may benefit the post-engrafted survival of MSCs and their paracrine potential to improve the therapeutic efficiency of MSC transplantation in neuropathic pain.

## Materials and methods

### Animals and surgical procedure of the CCI model

All animal experiments were performed in accordance with the guidelines of the Pusan National University Institutional Animal Care and Use Committee (IACUC: PNU-2021-0063). Adult Balb/C male and female mice weighing 20–25g were housed under a standard 12-h light/dark cycle with a regulated ambient temperature of 22–24°C. Mice were anesthetized with a single intraperitoneal injection of 2% Avertin (200 mg/kg) in accordance with the methods described in IACUC policy (The preparation, storage, and use of Tribromoethanol (Avertin) in mice). The CCI to sciatic nerve was used to induce the neuropathic pain as previously reported (16). Under aseptic conditions, the sciatic nerve was exposed by a skin incision along the femur, followed by the separation of biceps femoris muscles and superficial gluteal muscles without damaging the muscle bundles. The CCI was then induced by placing one, two, four ligatures (4/0 silk suture, 1 mm apart) around the nerve. The animals were returned to their cages following wound closure to recover, and the von Frey test was performed every 3–4 days for all groups.

### Isolation of tonsil-derived mesenchymal stem cells (TMSCs)

TMSCs were isolated as previously described (Doi: 10.3390/cells9010089). Briefly, tonsils were obtained from patients with chronic tonsillitis who had undergone tonsillectomy. To isolate stem cells, tonsil tissues were chopped using an autoclaved blade and scissors. Next, the tissues were digested in Type1 collagenase (Sigma-Aldrich, C9891) and hyaluronidase (Worthington Biochemical Corporation, LS002592) at 37°C for 1 h. The enzyme was neutralized with a 10% fetal bovine serum (FBS)-containing medium, and the samples were centrifuged at 3000 rpm for 10 min. After centrifugation, the samples were filtered through a 40-μm nylon mesh to remove debris and diluted in culture media for overnight incubation at 37°C under 5% CO_2_ conditions.

### Differentiation of TMSCs to adipocytes, osteoblasts, and chondrocytes

To induce the differentiation of MSCs to osteoblasts and adipocytes, TMSCs were seeded on 0.1% gelatin-coated 6-well plate at a density with 1 × 10^5^ cells. The osteogenic and adipogenic differentiation of TMSCs were induced by using StemPro osteogenesis differentiation kit (Thermo Fisher, A1007201) and StemPro adipogenesis differentiation kit (Thermo Fisher, A1007101), respectively. To induce the chondrogenic differentiation, pellets containing 5 × 10^4^ TMSCs were formed in 96-well polypropylene plates *via* centrifugation for 5 min at 300 × *g*. Cells were maintained in chondrogenesis media (StemPro chondrogenesis differentiation kit, A10071-01) at 37°C with 5% CO_2_. The differentiation induction media were changed every other day. Osteogenic differentiation of TMSCs was visualized by staining with Alizarin Red (Sigma Aldrich, TMS008) after 7 days of induction. The differentiation of TMSCs to adipocytes was demonstrated by staining the cytoplasmic inclusion of lipid droplet with Oil Red O (Sigma Aldrich, MAK194) at day 7. Toluidine Blue stain (Sigma Aldrich, 89640) was used to examine the formation of chondrogenic structure after 14 days under induction medium. The stained cells were observed on the microscope.

### MSC monolayer (2D) and spheroid (3D) cell cultures

TMSCs were cultured in gelatin-coated dishes containing α-MEM (Invitrogen, 12000022), 10% FBS (Invitrogen, 16000044), and 1% penicillin/streptomycin (Invitrogen, 15140122) for maintenance and 2D cell culture. To establish MSC spheroids, cells were harvested and cultured by hanging-drop methods at 1000, 2500, 5000, 10000, and 25000 cells/spheroid in a 150 mm *petri* dish and incubated for 3 days at 37°C under 5% CO_2_. After 3 days, the spheroids were collected from the dishes.

### Live and dead cell counting

MSC spheroids were collected from the plate and fragmented to single-cell conditions using Accutase. The enzyme-treated cells were neutralized with an FBS-containing medium. Neutralized cells were centrifugated at 1000 rpm for 4 min, and the pellets were diluted in 1 ml media. 10 µl of cell-contained media was mixed with 10 µl of trypan blue for staining of dead cells, and cell viability was assessed using a LUNA cell counter (LUNA-II Automated Cell Counter, Logos Biosystems, L40001).

### Cell transplantation

For cell transplantation, 1 × 10^6^ cells were suspended in the solution of Matrigel (1:20 dilution in the α-MEM, Corning, BC-354230) to obtain 1 × 10^6^ cells/100 µL for the 2D condition and 100 spheroids/100 µL for the 3D condition. For MSC transplantation, either single cells or spheroids were vertically injected into the thigh muscle region of CCI-induced mice using a 0.5 ml Hamilton syringe with 26-gauge needle. The injection was maintained at a slow flow rate (about 100 μL/min) and the needle was kept in place for at least 2 min post-injection to avoid leakage of the injecting solution. To prevent cell death by host defense, mice were treated with cyclosporine A (Sigma-Aldrich, 30024), an immunosuppressive drug, at a dose of 10 mg/kg every other day.

### Immunohistochemistry

The gastrocnemius muscle with sciatic nerves were fixed in acetone and incubated overnight at -20°C. Fixed tissues were washed and incubated in sucrose solution at 10%, 20%, and 30% dosage over 4 h. Tissues were embedded in O.C.T compounds and stored at -80°C to prepare cryomolds. Molds were sectioned to 20-μm and stored at -80°C. Sectioned samples were deparaffinized by cooling with acetone and blocked with M.O.M. IgG blocking reagents (Vector Laboratories, BM000-2202). For macrophage and inflammation staining of normal, 2 and 4 ligations, anti-CD68 (1:200, Bio-Rad, MCA 1957GA) and anti-Iba1 (1:200, Cell signaling, 17198S) were stained with DAPI. To measure neuroinflammation by CCI, anti-CD68 (1:200, Bio-Rad, MCA 1957GA) was co-stained with anti-human nuclei (1:200, Merck, MP-MAB1281) antibody. Primary antibodies were incubated overnight at 4°C. The sections were washed with PBS three times, and stained with goat anti-rat Alexa Fluor 488 dye (1:200, Invitrogen, A11006) for human nuclei antibody and goat anti-mouse Alexa Fluor 568 dye (1:200, Invitrogen, A11004) for CD68 or Iba1 antibody at room temperature for 2 h. The stained tissues were mounted using an anti-fade mounting solution containing DAPI for counter-staining of nuclei and visualized using the EVOS M5000 imaging system.

### Enzyme-linked immunosorbent assay (ELISA)

On day 9 after cell transplantation, all mice were anesthetized with 2% Avertin and 0.5~0.8 ml of blood was obtained directly from mouse hearts using a heparin-coated 1 mL syringe with 26-gauge needle. After collection, blood samples were centrifugated at 1000 g for 30 min to isolate sera. The levels of TNF-α and INF-γ in serum were measured by using ELISA assay kits (TNF-α: Biolegend, 430904 and IFN-γ: Biolegend, 43804). Briefly, TNF-α and IFN-γ antibodies (diluted 1:200) were coated on 96-well non-coated plate overnight at 4°C. The plate was then washed four times and blocked for 1 h at room temperature. After washing four times, sera were added to a plate and incubated for 2 h. Sera-incubated plates were washed four times to remove overexpression and reacted with detection antibodies (diluted 1:200) at room temperature. After 1 h, the plate was washed four times, treated with horseradish peroxidase (HRP) (diluted to 1:1000) for 30 min at room temperature, and washed five times to remove residual HRP. The washed plates were incubated with HRP substrate solution for 15 min before stopping the reaction with the stop solution. The plate was analyzed using a microplate reader (TECAN Sunrise, TECAN Life Science).

### Staining of 2D and 3D MSCs using *in vivo* tracker

MSCs were stained using an *in vivo* tracker (IVISense 680 Fluorescent Cell Labeling Dye; PerkinElmer, NEV12000). Briefly, cells were harvested from the culture dishes and stained with an *in vivo* tracker at a dosage of 2.5 × 10^8^/ml, for 15 min of 2D MSCs and 30 min of 3D MSCs, at room temperature, protected from light. The stained cells were washed three times in PBS and diluted in Matrigel for cell transplantation. The cells were vertically transplanted close to the sciatic nerve using a 26-gauge syringe needle. To confirm the bioluminescence levels of the transplanted cells, mice were visualized using an *in vivo* imaging system (CRI Maestro) to measure cell engraftment and viability 24 h, 1 week, and 2 weeks after cell transplantation.

### Von Frey test (PWT)

To confirm mechanical allodynia, the PWT was measured using the von Frey test. CCI-induced mice were placed on a stainless mesh and adapted for 10 min. After adaptation, von Frey filaments (JEUNG DO BIO & PLANT, JD-SI-11F) were stimulated in the hind paws. Von Frey filaments were selected from 0.16 g, and the filament force was gradually increased until the paw was raised. To select mice for CCI modeling, the PWT was measured three times at 2-day intervals, and the 1.0 g value mice were selected before surgery. After surgery, the PWTs were recorded every 3 days and quantified at the filament force levels of the paw reaction.

### Target gene qPCR screening

To extract total RNA, the injured sciatic nerve was isolated from all groups, and RNA was separated using TRIzol reagent (Sigma-Aldrich, T9424) according to the manufacturer’s protocols. The RNA concentration was measured using a NanoDrop spectrophotometer. RNA was reverse transcribed with oligo dT primers using Superscript II. For target gene screening, we performed real-time quantitative RT-PCR using SYBR Green PCR Master Mix (Applied Biosystems, ABS-4309155). PCR amplification was performed using the inflammatory response and autoimmunity gene-specific primers (Accutarget ™ qPCR Screening kit, Bioneer, SH-000-10 for human origin; SM-000-10 for mouse origin) as follows: 40 cycles of denaturation at 95°C for 5 s, annealing at 58°C for 25 s, and elongation at 72°C for 30 s. Target gene expression was determined by normalization to endogenous GAPDH or actin using the comparative cycle time method.

### Statistical analysis

Statistical differences between groups were analyzed using the t-test and one-way analysis of variance (ANOVA) for multiple comparisons. T-test was performed two-tailed Student’s t-test and one-way ANOVA was performed using Tukey’s posthoc test. All values are presented as mean ± standard error of the mean, and the p-values are indicated as follows: * *P* < 0.05, ** *P* < 0.01, *** *p*< 0.005 and **** *p*< 0.001. All statistical analyses were performed using GraphPad Prism 9 (GraphPad Software, Inc.).

## Results

### Induction of the optimal CCI pain model by two ligation knots

**Figure 1:**
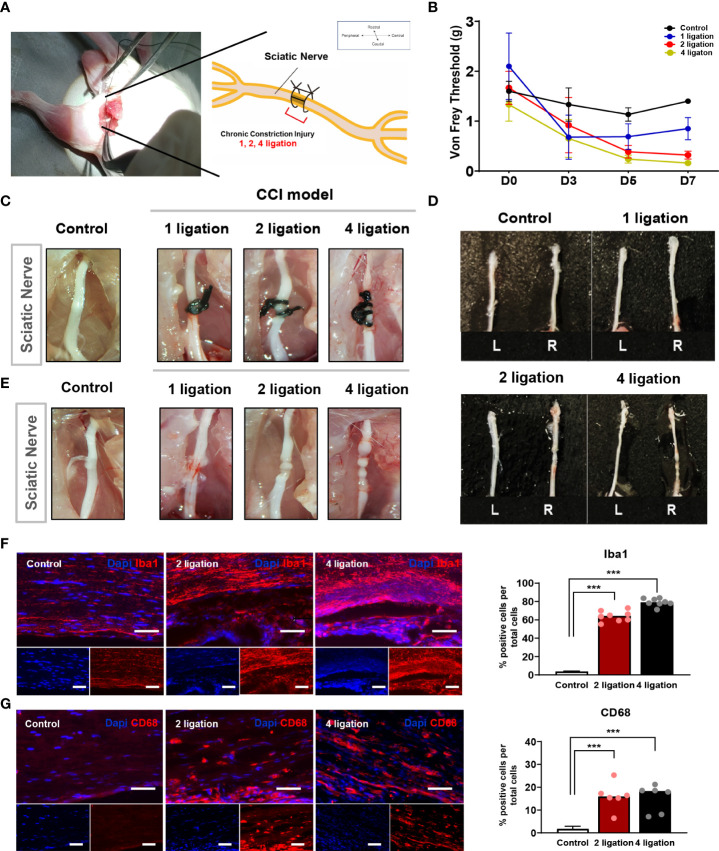
Induction of chronic constriction injury mouse model and inflammatory factors. **(A)** The location of ligation knots on the right hindlimb sciatic nerve. **(B)** Von Frey test of 1, 2, 4 ligations CCI mouse model. Day 0 is baseline threshold before the injury (n = 5 mice for each group). Morphological features of **(D)** sciatic nerve **(C)** before and **(E)** after ligations. **(F)** Immunofluorescence staining of Iba-1 positive cells (Red) with DAPI (Blue). Percentage of Iba-1 positive cells in 2 and 4 ligations CCI model. Scale bar: 150 μm. **(G)** Immunofluorescence staining of CD68-positive cells (Red) and DAPI (Blue). Percentage of CD68-positive cells in 2 and 4 ligations compared to control. Scale bar: 150 μm. Data are presented as mean ± SEM. Statistical differences were analyzed using one-way ANOVA, followed by Tukey’s *post hoc* test, ***p< 0.005.

The CCI animal model is a well-established neuropathic pain model reported previously (17–19) and is particularly useful for quantitative assessment of the therapeutic efficacy of cell transplantation on chronic neuropathic pain. To apply a suitable CCI animal model for cell therapy, we first validated a ligation-knot-based constriction injury generated by one, two, or four ligations on the partial sciatic nerve of the right hindlimb (**Figure 1A**). To measure the nerve-induced pain response, we performed the von Frey test in each mouse every 2 days. The von Frey test assesses mechanical pain response-like behavior and measures the paw withdrawal threshold (PWT) by flicking its paw away from the filament force. As shown in **Figure 1B**, before the injury, the baseline (D0) threshold, the PWT, was not significantly different among the groups before modeling. However, after surgery applying the ligation, the PWT decreased significantly in the groups with either two or four ligations compared to the control group, and the group with one ligation spontaneously recovered at 7 days compared to day 3 after surgery (one knot: 0.85 ± 0.44 g; two knots: 0.32 ± 0.13 g; four knots: 0.22 ± 0.12 g). In addition to the paw response to ligation, we examined the involvement of inflammatory markers. In all groups, the suture knots were carefully removed under a microscope (**Figures 1C–E**), followed by staining with anti-CD68 and anti-Iba1 antibodies. The group with one ligation exhibited mild inflammation in the middle part of the nerve but no strong CD68 expression. Consistently, both the two and four ligation groups exhibited a strong increase in the number of Iba1-positive cells and moderate increase of CD68-positive macrophages in tissues (**Figures 1F, G**; **Supplementary Figure 1**). This finding suggests that either two or four ligations can induce hyperalgesia and produces the CCI neuropathic pain phenotype.

**Figure 2:**
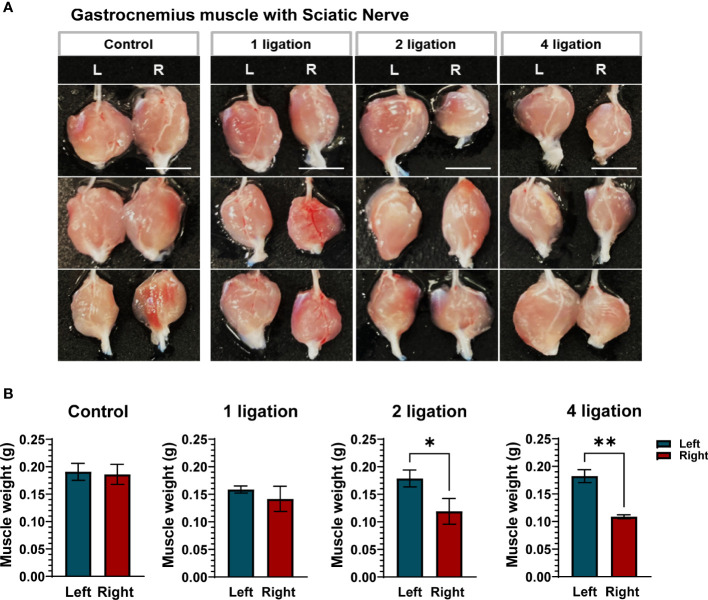
Gastrocnemius muscle weight according to the ligations. **(A)** Gastrocnemius muscles on control, 1, 2 and 4 ligations. Scale bar: 1 cm. **(B)** The muscle weight (g) was estimated as a ratio to compare the left hindlimb muscle (Blue bar) to the right contralateral site (Red bar) (n = 5 per group). Data are presented as mean ± SEM. Statistical significance was analyzed using two-tailed Student’s t-test, *p < 0.05, ** *p*< 0.01.

Subsequently, the weight of the gastrocnemius muscle was validated in the CCI model. The muscle weight (g) of all groups was estimated as a ratio to compare the left hindlimb muscle to the right contralateral site. Partial gastrocnemius muscle atrophy was detected in both the two and four ligation groups, as they exhibited a significant loss (approximately 30–40%) of muscle weight compared to the control and one ligation groups (**Figure 2B**). Taken together, these results indicate that either two or four ligations induced significant muscle atrophy and moderate nerve damage in mice compared with one ligation and that four ligations group mice had increased severe nerve damage, such as sensory loss (**Figure 2A** and **Supplementary Figure 1**). Therefore, according to the results of the ligation test, we selected two ligations to generate the CCI pain mouse model for this study.

### MSC spheroids with a density of 10000 cells are suitable for cell transplantation

Human tonsil-derived mesenchymal stem cells (TMSCs) were isolated from patient tonsil tissues and digested into small clumps. These clumps appeared heterogeneous at passage 0 (P0) and expanded through progressive subculture. By passages 3 and 4, the cells developed a typical MSC-like morphology with elongated and heterogeneous single cells. The surface phenotypes of MSCs were analyzed by flow cytometry analysis. TMSCs were positive for several MSC-specific cell surface markers, including CD105, CD90, and CD44 (**Supplementary Figure 2A**), and they were negative for hematopoietic cell markers, such as CD34 and CD45 (data not shown). TMSCs also had the capacity to differentiate into tri-lineage cell types including adipocytes, osteoblasts, and chondrocytes (**Supplementary Figure 2B**). Not only 2D MSCs but also 3D MSC spheroids expressed the three MSC markers (**Supplementary Figure 2C**). Therefore, these results suggest that TMSCs exhibit MSC phenotype and multipotent differentiation potential.

**Figure 3:**
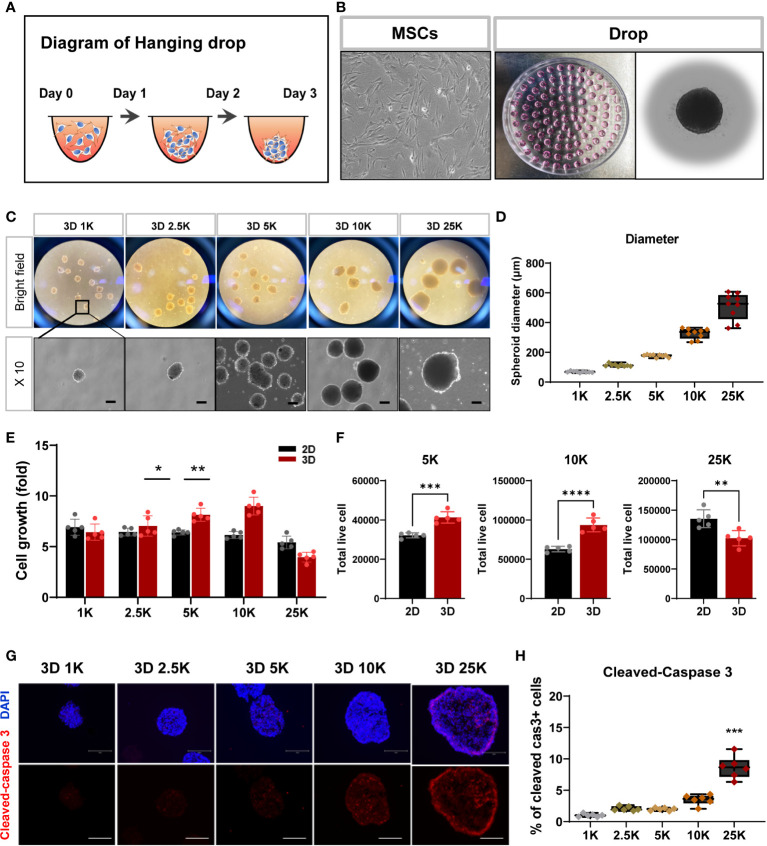
Structural and cell viability of tonsil-derived MSC spheroids (3D) depending on cell densities. **(A, B)** Suspension culture of MSC spheroids using hanging drop method. **(A)** Schematic diagram of hanging drop. **(B)** Bright-field images for tonsil-derived MSCs (TMSCs) and drops in a *petri* dish. **(C)** Morphological features of the spheroids formed at five different cell densities. Spheroids incubated for 3 days and then collected them from the *petti* dish. Scale bar: 100 μm. **(D)** The diameter of spheroids (μm). **(E)** Fold change of spheroid cell growth. **(F)** The number of live cells in a density of 5000, 10000 and 250000 cell spheroids. Spheroids were dissociated into single cells and counted the total viable cells by trypan blue assay. **(G)** Immunofluorescence staining of cleaved-caspase 3 positive cell (Red) with DAPI (Blue) according to spheroids. Scale bar: 100 μm. **(H)** Quantification of the expression of cleaved-caspase 3. The high-density spheroid was increased the cleaved-caspase positive cells compare to smaller spheroids. Data are presented as mean ± SEM. Statistical significance for spheroid diameters, spheroid cell growth and % of cleaved caspase3 cells were analyzed using one-way ANOVA, followed by Tukey’s *post hoc* test, *p < 0.05, **p < 0.01, ***p < 0.005. Statistical significance for total live cells was analyzed using two-tailed Student’s t-test, **p < 0.01, ***p < 0.005, ****p < 0.001. Cell densities are 1K, 1000 cells; 2.5K, 2500 cells; 5K, 5000 cell; 10K, 10000 and 25K, 25000.

Numerous laboratories have investigated the applicability of spheroid MSCs for cell therapy (22, 23). Based on the findings that spheroids offer various advantages and can significantly enhance the viability of grafted cells, spheroids have recently been used more frequently than monolayer cultured cells for the purpose of cell transplantation. As the size of spontaneously formed spheroids varies between experiments, we sought to establish spheroids with a more suitable cell density and size for stem cell therapy. For spheroid aggregation, TMSCs were cultured in suspension using the hanging drop method (24–26) (**Figures 3A, B**), and spheroids were produced at five cell densities (1000, 2500, 5000, 10000, and 25000 cells). As shown in **Figures 3C, D**, all spheroids were visible to the naked eye after 1 day of culture and grew in size by day 3. The diameters of the formed spheroids ranged from 60 to 600 μm (1K: 68.9 ± 5.03 μm; 2.5K: 113 ± 8.91 μm; 5K: 176 ± 9.23 μm; 10K: 326 ± 35.2 μm; 25K: 510 ± 89.52 μm) depending on the cell densities, demonstrating that spheroid sizes can be precisely manipulated by the cell-seeding densities. Moreover, we counted the live/dead cells in all spheroids to determine the number of living cells in the final spheroid formed on day 3. To calculate cell viability and growth, we dissociated the spheroids into single cells using Accutase and performed trypan blue staining. The total number of live cells increased linearly with increasing cell numbers; however, with 25000 cells, the number of live cells was dramatically reduced (**Figures 3E, F**). Furthermore, to investigate apoptosis in spheroids of different sizes, the assembled spheroids were harvested and analyzed for apoptosis markers by immunostaining. Cleaved caspase 3-positive cells showed significantly increased in the high-density spheroids (up to 25000 cells) compared to that observed in smaller spheroids and was mostly expressed on the outer surface of the spheroids (**Figures 3G, H**).

As an extension of the previous experiment, the inflammatory environment in the injured region may affect survival after transplantation, and spheroid cell damage may occur first during the application of the injection needle. To investigate whether spheroids of various sizes exhibited alterations in their surface shape after syringe application, all spheroids were passed through a 26-gauge syringe with a needle. The surface of small spheroids (< 10000 cells) appeared to have a grainy texture and exhibited a light-silver sheen compared to that of large-sized spheroids. Moreover, larger spheroids (> 10000 cells) exhibited wall damage during flow through a syringe needle (**Supplementary Figure 3**). We did not check for surface markers, but variations in the formation of spheroids with a characteristic spherical shape were observed, as shown in **Supplementary Figure 3**. Thus, we suggest that 10000 cells for spheroids are suitable for cell transplantation in this study and highlight the importance of determining spheroid size/cell density to obtain consistent and reliable results of cell transplantation in animal models of disease.

### MSC spheroids exhibit improved efficiency of cell survival at the target site and afford pain relief and downregulation of inflammatory factors

**Figure 4:**
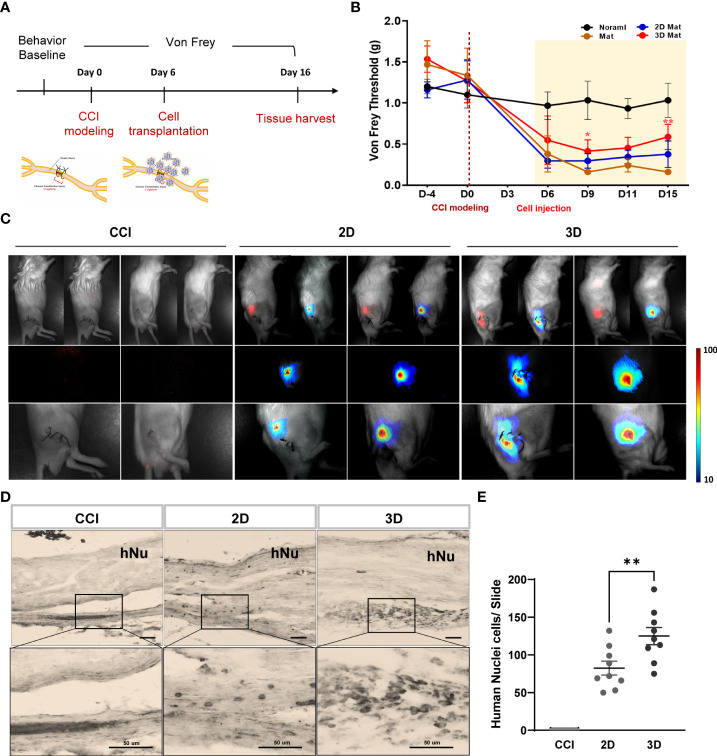
Von Frey test and grafted cell survival of MSC spheroids compared to monolayer cells. **(A)** Schematic overview of MSC spheroids and monolayer cell transplantation. All experimental groups are normal, vehicle, monolayer (2D MSC) and spheroid (3D MSC) (n = 6 mice for each group). Before CCI modeling (D-4), all groups were measured the baseline threshold of von Frey test. On day 6, vehicle and cell transplanted groups (1 X 10^6^ cells/100ul for 2D and 100 spheroids/100ul for 3D) were transplanted into the injured hindlimb sciatic muscle region. All groups were performed the von Frey test every 3~4 days and sacrificed them on day 15. **(B)** Von Frey test of normal, vehicle, 2D MSC and 3D MSCs. **(C)** *In vivo* NIR-2 fluorescence whole body imaging. The grafted cells are brightly labeled and detected on the injured sciatic nerve area. DAB immunohistochemistry staining **(D)** and number of positive cells **(E)** using an antibody against human nuclei (hNu) in the sciatic nerve. Scale bar: 50 μm. Von Frey threshold curves were statistically analyzed by two-way ANOVA. Data are presented as mean ± SEM, **p < 0.01. Statistical differences for the human nuclei cells were analyzed using one-way ANOVA, followed by Tukey’s *post hoc* test, **p< 0.01.

Based on the optimal spheroid size and cell density established in this study, we examined the *in vivo* effects of optimized spheroid MSCs (10000 cells) against neuropathic pain. Mice were randomized into the following four groups: normal, vehicle, 2D MSCs, and 3D MSCs. CCI neuropathic pain was induced in all mice, except for the normal group, *via* two ligations of the sciatic nerve. Before CCI surgery, we performed von Frey tests and observed that the baseline threshold in all groups was 1.2–1.6 g. After surgery, the von Frey thresholds of the CCI-induced groups significantly decreased in between day 0 and day 6 relative to the baseline normal group, consistent with the result of **Figure 1B**. On day 6, we prepared vehicle and cell groups (1 × 10^6^ cells/100 µL for the 2D condition and 100 spheroids/100 µL for the 3D condition) and transplanted them into the sciatic muscle area of the injured hindlimb. The von Frey test was performed every 3–4 days (**Figure 4A**). Compared to baseline, the vehicle group showed significant mechanical allodynia and gradually decreased von Frey thresholds. Conversely, cell injection, especially in the 3D condition, significantly improved the pain response from 0.16 to 0.8 g on day 15 and gradually increased the von Frey threshold from 9 days compared to the 2D condition (**Figure 4B**). This suggests that MSC spheroids are sufficient to ameliorate the impaired paw response because of their good graft survival.

**Figure 5:**
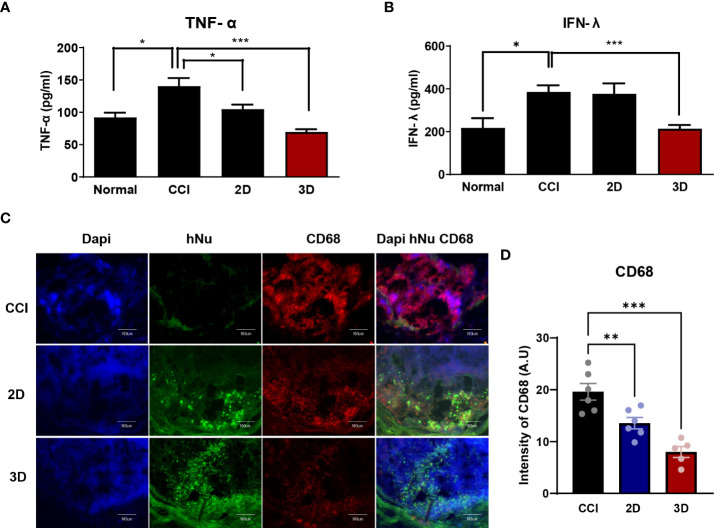
The expression of inflammatory factors in CCI, MSC 2D and 3D graft sites. Pro-inflammatory cytokine tumor necrosis factor- α (TNF-α) **(A)** and interferon- γ (IFN-r) **(B)** level in normal, CCI and cell transplantation groups. Cytokine concentrations are indicated as pg/ml (n = 6 per group). **(C)** Immunofluorescence staining of CD68 positive cells (Red) and human nuclei positive cells (hNu; Green) double staining with DAPI (Blue) in the cell transplantation region. Scale bar: 100 μm. **(D)** Quantification of CD68 intensity in the CCI and cell transplantation groups. (n = 6 per group). The area and fluorescent intensities of all CD68 positive cells in each cell grafted regions measured by Image J. Intensity is indicated as arbitrary unit (A.U.). Scale bar: 100 μm. Data are presented as mean ± SEM. Statistical differences for the intensities of CD68 expression were analyzed using one-way ANOVA, followed by Tukey’s *post hoc* test, *p < 0.05, **p < 0.01, ***p < 0.005.

To support the previous data, we evaluated the distribution of the transplanted 2D and 3D MSCs. Monolayer and spheroid MSCs labeled with an *in vivo* tracker (27) were directly injected into the gastrocnemius muscle close to the sciatic nerve, and measurements were performed using optical fluorescence imaging at 680 nm after cell transplantation. Interestingly, on day15, the results showed that 3D MSCs persisted well in the target region and yielded a signal intensity in grafted cells that was significantly greater than that detected in 2D MSCs (**Figure 4C**). The stereological estimates of surviving human nuclei cells show an average of 83 ± 27 cells and 125 ± 34 cells in 2D and 3D graft site close to the sciatic nerve (**Figures 4D, E**). As it is possible to establish correlations with inflammation signaling through macrophages, we harvested the gastrocnemius muscle and performed staining for the pan-macrophage marker CD68, which triggers the release of cytokines *via* macrophage infiltration. As shown in **Figures 5C, D**, the groups with CCI injury exhibited high expression levels of CD68 in the mid-nerve region, while the 2D and 3D MSC injection groups showed significantly reduced expression of CD68. Therefore, our results demonstrated that spheroids have better survival at the engrafting site compared to monolayer cultured MSCs and alleviates hyperalgesia induced by nerve damage by reducing macrophage infiltration. Next, we screened for inflammation-response-related genes in the cell transplantation groups, including the control group, for further analysis of a key mechanism that reduces inflammation.

### Good graft survival of MSC spheroids modulates the host inflammatory response induced by sciatic nerve injury pain

After nerve injury, proinflammatory cytokines such as tumor necrosis factor- α (TNF-α), interferon- γ (IFN-γ), and interleukin-6 are upregulated in CCI animal models and are important effectors in modulating immune responses (12, 28–30). In this context, we collected blood from six mice in each group on day 9 after cell injection and assessed TNF-α and IFN-γ levels in the isolated serum. As shown in **Figures 5A, B**, we observed a significant difference between CCI and spheroid groups as follows: the TNF-α levels in the CCI, 2D MSC, and 3D MSC groups were 130.1 ± 18.1, 126.6 ± 25.3, and 61.8 ± 3.6 pg/ml, whereas IFN-γ levels were 386 ± 67.8, 366.5 ± 89.9, and 213.3 ± 53.5 pg/ml, respectively. As a result, the plasma TNF-α levels in the spheroid group decreased by approximately 47% and the IFN-γ levels by approximately 55% compared to the CCI group. Hence, MSC spheroids exerted a good survival effect, which dramatically reduced the levels of proinflammatory cytokines.

**Figure 6:**
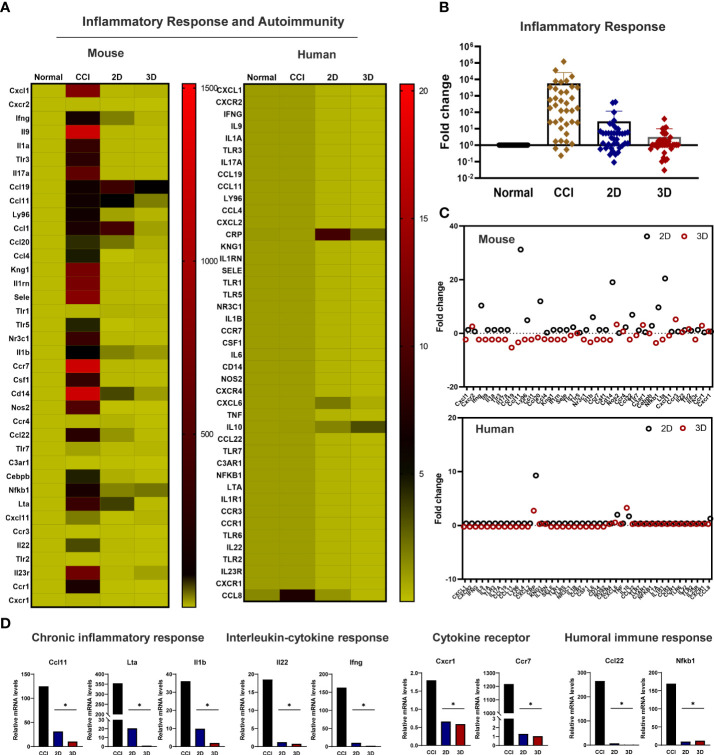
Modulation of the inflammatory response genes in the transplanted MSC spheroid and monolayer cells. **(A)** Genetic heatmap analysis revealed that 30 genes were associated with mouse- and human-specific inflammatory genes. Primers were obtained from Accutarget ™ qPCR Screening kit (Bioneer). Target gene expression was determined by normalization to endogenous GAPDH or actin using the comparative cycle time method. **(B)** Fold change of whole inflammatory response genes related to mouse-specific response in CCI and cell transplantation groups. **(C)** Scatter plot of mouse and human-specific inflammatory response genes. **(D)** In the comparison of 3D and 2D MSCs, mainly reduced the expression of chronic inflammatory response genes (*Ccl11* (Eotaxin), *Il1a* and *Lta*), interleukin–cytokine signaling genes (*Ifng*, *Ilb*, and *Il22*), cytokine receptor genes (*Ccr7* and *Cxcr1*), and humoral immune response genes (*Ccl22*, *Il1b*, and *Nfkb1*) (n = 4 per group). Data are presented as mean ± SEM. Statistical differences were analyzed using one-way ANOVA, followed by Tukey’s *post hoc* test, *p < 0.05.

To further explore the molecular mechanism underlying the peripheral inflammation observed within the injured nerve after cell transplantation, we analyzed the expression of genes involved in the host inflammatory response (**Supplementary Table 1**). The injured sciatic nerves isolated from the CCI, 2D MSC, and 3D MSC groups, including the control and inflammatory response genes, were examined *via* qPCR screening analysis. Genetic heatmap analysis revealed that although 30 genes related to mouse-specific inflammatory signaling were upregulated in the CCI group compared to the normal group, these genes were significantly downregulated in both the 2D and 3D groups (**Figure 6A**). Moreover, human-specific inflammatory genes were not expressed in the CCI group, and a few of these genes were upregulated in the 2D and 3D groups (**Figures 6A, C**). Finally, a comparison of 3D and 2D MSCs revealed that the overall expression of mouse inflammatory genes decreased to a greater extent in the 3D condition (**Figure 6B**). The engrafting of 2D and 3D MSCs mainly reduced the expression of interleukin–cytokine signaling genes (*Ifng*, *Ilb*, and *Il22*), cytokine receptor genes (*Ccr7* and *Cxcr1*), and humoral immune response genes (*Ccl22*, *Il1b*, and *Nfkb1*) (**Figure 6D**). Interestingly, compared to other signaling, the main genes involved in the functional signaling of chronic inflammatory responses, such as *Ccl11/Eotaxin*, *Il1A*, *Lta*, and *Tnf*, were greatly downregulated under 3D groups. These finding indicates that, under conditions of neuropathic pain, the chronic inflammatory response genes may be key regulators to reduce the pain response and improve motor behavior after cell transplantation. Therefore, we suggest that transplantation of MSC spheroids has a sufficient effect in reducing and modulating inflammation-related genes triggered by nerve injury pain *via* chronic inflammatory response signaling.

## Discussion

The CCI model is a simple means of replicating chronic pain, although the histological and behavioral results of nerve ligation vary (17–19). Therefore, it is necessary to examine the ligations regarding the effects of consistent compressive pain. To this end, we first investigated a suitable number of ligation knots on the sciatic nerve on the right hindlimb. All groups underwent von Frey testing, which measures the paw response to the application of filament force. The results showed that both two and four ligations induced hyperarlgesia and produced the CCI neuropathic pain phenotype. Consistent with the pain response, two ligations produced muscle atrophy/weakness and moderate nerve damage compared to one ligation, whereas four ligations resulted in severe nerve damage, such as sensory loss and hyper-inflammation. Therefore, we selected two ligation knots to generate a CCI neuropathic pain mouse model.

Many studies on the treatment of neuropathic pain using stem cells have shown promising results in preclinical research, with MSCs being the best tool for cell-based therapy (8–10). However, the important aspects of the mechanism and signaling underlying that occur the injured regions after MSC transplantation remain unclear. Next, for spheroid aggregation, we cultured MSCs using the hanging drop method, as shown in **Figure 3A** (24–26). Previous studies have reported the formation of spheroids of varying sizes using this method. Importantly, the aggregation size must be carefully considered because of the limitations in the diffusive length of nutrient transport. Waslberg et al. showed that the core of spheroids with radii > 200 µm is vulnerable to hypoxia, with cell death and breaking, during application using a syringe and needle (15, 31–33). Thus, we examined the range of 1000 to 25000 cells per spheroid and observed the diameter of this range. In our experiment, spheroids of approximately 10000 cells had a mean radius of approximately 200 µm and were the most viable, as assessed based on caspase 3 activity, compared to spheroids of larger sizes. Moreover, when 10000 spheroids were passed through a 26-gauge syringe needle, their surface was maintained more consistently than that of the larger spheroids. Based on this result, we aggregated 3D spheroids at a density of 10000 cells and investigated their *in vivo* effects on CCI neuropathic pain.

MSC spheroids elicit neuroprotective and anti-inflammatory effects, which are known MSC therapies in disease models (11–14). However, the detailed mechanisms underlying the effects of spheroids on neuropathic pain remain unclear. For cell transplantation, spheroid MSCs and monolayer MSCs at a density of 1 × 10^6^ cells were transplanted close to the sciatic nerve. The pain response was measured by the von Frey test. In the spheroid group, 3D MSCs exhibited a significantly improved pain response. Furthermore, we found that engrafted 2D MSCs with phosphate-buffered saline (PBS) spread out to other regions and migrated to different target sites, whereas 3D MSCs with PBS persisted well at the grafting site (data not shown). This demonstrated that 3D spheroids have better survival at the target site than 2D MSCs and may alleviate hyperalgesia. To confirm this finding, we investigated the levels of proinflammatory cytokines triggered by inflammation derived from host immune cells and their effect on cell survival. The levels of TNF-α and IFN-γ released into the plasma were greatly reduced (by approximately 50%) in 3D MSCs, indicating that the inflammatory response is regulated at the engraftment site. Overall, immediately after nerve damage, immune cells are activated to release cytokines and chemokines, followed by macrophage infiltration into the damaged region (within hours to days) (34–37). Here, we identified 30 genes associated with the upregulation of the mouse-specific inflammatory response in the CCI group, as well as genes that were significantly downregulated in interleukin-cytokine signaling, cytokine signaling (38), and the humoral immune response in 2D and 3D MSCs (39). Importantly, we found that implantation of MSC spheroids was modulated by chronic inflammatory response signaling to reduce inflammation.

In this study, we established a suitable animal model of CCI neuropathic pain and observed the good survival potential afforded by MSC spheroid injection and the good effects of the injected MSC spheroids on the repair of pain defects compared to the monolayer cell condition. We demonstrated that MSC spheroids containing 10000 cells exhibited greater resistance to apoptosis and significantly reduced secretion of immune response factors induced by CCI. Furthermore, good graft survival of spheroids dramatically modulated proinflammatory cytokines and genes related to chronic inflammatory response, suggesting that MSC spheroids may enhance the alleviation of pain and motor function. These data provide strong evidence that spheroids have the potential to facilitate MSC-based cell therapies for the repair of induced neuropathic pain injury.

## Data availability statement

The original contributions presented in the study are included in the article/**Supplementary Material**. Further inquiries can be directed to the corresponding author/s.

## Ethics statement

The studies using human tonsils were reviewed and approved by the Institution Review Board of Pusan National University Hospital (1801-033-062). The patients/participants provided their written informed consent to participate in this study. The animal study was reviewed and approved by Institutional Animal Care and Use Committee of Pusan National University.

## Author contributions

NL designed the study and wrote the manuscript. GP wrote the manuscript and performed the experiments. JL performed the experiments. EC, HM, DK, and SC analyzed the data. YS obtained the TMSCs. JK conceived and supervised the project. All authors contributed to the article and approved the submitted version.

## Funding

This work was supported by the National Research Foundation of Korea (NRF) grants funded by the Korean government (MISIT) (NRF-2021R1C1C201330311; NRF-2020R1A2C2011654).

## Acknowledgments

We wish to thank all the members of the Gene and Cell Therapy Research Laboratory in the Department of Physiology who participated in this cell therapy project.

## Conflict of interest

The authors declare that the research was conducted in the absence of any commercial or financial relationship that could be construed as a potential conflict of interest.

## Publisher’s note

All claims expressed in this article are solely those of the authors and do not necessarily represent those of their affiliated organizations, or those of the publisher, the editors and the reviewers. Any product that may be evaluated in this article, or claim that may be made by its manufacturer, is not guaranteed or endorsed by the publisher.
