# Supplementary material for: Mesenchymal stem cell spheroids alleviate neuropathic pain by modulating chronic inflammatory response genes
Source: Front Immunol. 2022 Aug 8;13:940258. doi: 10.3389/fimmu.2022.940258 (PMC9393760; doi:10.3389/fimmu.2022.940258)
Supplement: Supplementary file 2 [file Table_1.docx]

**Supplemental Table**

**Table 1. The inflammatory response & Autoimmunity genes**

| Group & signaling | Function | Gene |
| --- | --- | --- |
| **Cytokine (Chemokines)** | Communication between innate and adaptive immune cells | *CCL2, CCL3, CCL4, CCL5, CCL7, CCL8CCL11, CCL13, CCL16, CCL17, CCL19, CCL21, CCL22, CCL23, CCL24, CXCL1, CXCL2, CXCL3, CXCL5, CXCL6, CXCL9, CXCL10,* |
| **Cytokine (Interleukins)** | Role of cytokines in mediating communication between immune cells | *IL1A, IL1B, IL1RN, IL5, IL6, IL10, IL15, IL17A, IL18, IL22, IL23A, CXCL8, CSF1, LTB, TNFSF14* |
| **Cytokine Receptors** | IL-1 and IL-10 signaling | *IL1R1, IL1RAP, IL6R, IL10RB* |
| **Chemokine Receptors** | Chemokine signaling | *CCR1, CCR2, CCR3, CCR4, CCR7, CXCR1, CXCR2, CXCR4* |
| **Cytokine mediated signaling** | Cytokine signaling | *CCL2, CCL5, CCR1, CCR2, IFNG, IL1, IL1B, IL1R1, IL5, IL6, IL6R, MYD88, TNF* |
| **Acute phase response** | Acute phase response signaling | *CEBP, CRP, PTGS2* |
| **Chronic inflammatory response** | Differential regulation in macrophages and dendritic cells | *CCL5, CCL11, IL1B, LTA, TNF* |
| **Humoral immune response** |  | *CCL2, CCL3, CCL7, CCL16, CCL22, CCR2, CCR7, CD40, IL10, IL18, IL1B, IL6, ITGB2, NFKB1* |
| **Regulation of the inflammatory response** | Regulation of inflammatory response | *BCL6, CD14, CD40LG, FOS, IL9, NOS2, NR3V1, TLR3, TLR6, TLR7* |
