# Supplementary material for: Mesenchymal stem cell spheroids alleviate neuropathic pain by modulating chronic inflammatory response genes
Source: Front Immunol. 2022 Aug 8;13:940258. doi: 10.3389/fimmu.2022.940258 (PMC9393760; doi:10.3389/fimmu.2022.940258)
Supplement: Supplementary file 1 [file DataSheet_1.docx]

**Supporting Information**

**Mesenchymal stem cell spheroids alleviate neuropathic pain by modulating chronic inflammatory response genes**

Nayeon Lee^1,2,#^, Gyu Tae Park^1,2,#^, Jae Kyung Lim^2^, Eun Bae Choi^2^, Hye Ji Moon^2^, Dae Kyoung Kim^2^, Seong Min Choi^2^, Young Cheol Song^2^, Tae Kyun Kim^3^, and Jae Ho Kim^1,2,¶^

^1^Convergence Stem Cell Research Center, Medical Research Institute, Pusan National University, Yangsan 50612, Gyeongsangnam-do, Republic of Korea; ^2^Department of Physiology, School of Medicine, Pusan National University, Yangsan 50612, Gyeongsangnam-do, Republic of Korea; ^3^Department of Anesthesia and Pain Medicine, Pusan National University Yangsan Hospital, Yangsan, 50612, Gyeongsangnam-do, Republic of Korea

**Supplemental Figures**

**Supplemental Figure 1. DAB staining of microglia and macrophage markers in injured sciatic nerve.** The morphology and location of macrophages are indicated with Iba-1 (A) and CD68 (B) staining. Scale bar: 100 μm.

**Supplemental Figure 2. Characterization of human tonsil-derived mesenchymal stem cells (TMSCs).** (A) Flow cytometry analysis of MSC phenotype. TMSCs were positive for various cell surface markers, including CD105, CD90 and CD44, and negative for CD34 and CD45 in both MSC spheroids and monolayer cells. (B) Tri-lineage differentiation of tonsil-derived MSCs. Induced TMSCs were stained by Alizarin Red, Oil Red O and Toluidine Blue to identify adipogenic, osteogenic, and chondrogenic differentiation, respectively. (C) Western blot analysis of MSC markers (CD166, CD105, CD44) and a hematopoietic cell marker (CD34) in 2D and 3D cultured MSCs. The cell numbers (1×10^3^ ~ 2.5×10^4^) used for formation of MSC spheroids are indicated.

**Supplemental Figure 3. Spheroid cell membrane damage after syringe-needle injection.** Bright images of the spheroids surface after application of the injection needle. All spheroids were passed through a 26-gauge syringe with a needle. Large size of spheroids, a density of 25000 and 50000 cells, exhibited wall damage compared to small size of spheroids.
